# The relationship between serum albumin levels and 24-h ambulatory blood pressure monitoring recordings in non-diabetic essential hypertensive patients

**DOI:** 10.6061/clinics/2016(05)03

**Published:** 2016-05

**Authors:** Elbis Ahbap, Tamer Sakaci, Ekrem Kara, Tuncay Sahutoglu, Yener Koc, Taner Basturk, Mustafa Sevinc, Cuneyt Akgol, Arzu O. Kayalar, Zuhal A. Ucar, Feyza Bayraktar, Abdulkadir Unsal

**Affiliations:** I Sisli Etfal Training and Research Hospital, Department of Nephrology, Istanbul, Turkey; II Recep Tayyip Erdogan University, School of Medicine, Rize Educational and Research Hospital, Internal Medicine, Nephrology, Rize, Turkey

**Keywords:** Hypertension, Serum Albumin, Ambulatory Blood Pressure, Dipping Status, Nocturnal Blood Pressure

## Abstract

**OBJECTIVES:**

The goal of this study was to evaluate the relationship between serum albumin levels and 24-hour ambulatory blood pressure monitoring (24-h ABPM) recordings in non-diabetic essential hypertensive patients.

**METHODS:**

A total of 354 patients (mean [SD] age: 55.5 [14.3] years, 50% females) with essential hypertension and 24-h ABPM recordings were included. Patient 24-h nighttime and daytime ABPM values, systolic and diastolic dipping status and average nocturnal dipping were recorded. The correlations between serum albumin levels and nocturnal systolic and diastolic dipping were evaluated, and correlates of average nocturnal systolic dipping were determined via a linear regression model.

**RESULTS:**

Overall, 73.2% of patients were determined to be non-dippers. The mean (SD) levels of serum albumin (4.2 [0.3] g/dL *vs*. 4.4 [0.4] g/dL, *p*<0.001) and the average nocturnal systolic (15.2 [4.8] mmHg *vs*. 0.3 [6.6] mmHg, *p*<0.001) and diastolic dipping (4.2 [8.6] mmHg*vs*. 18.9 [7.0] mmHg, *p*<0.001) were significantly lower in non-dippers than in dippers. A significant positive correlation was noted between serum albumin levels and both systolic (r=0.297, *p*<0.001) and diastolic dipping (r=0.265, *p*<0.001). The linear regression analysis revealed that for each one-unit increase in serum albumin, the average nocturnal dip in systolic BP increased by 0.17 mmHg (*p*=0.033).

**CONCLUSION:**

Our findings indicate an association between serum albumin levels and the deterioration of circadian BP rhythm among essential hypertensive patients along with the identification of a non-dipper pattern in more than two-thirds of patients. Our findings emphasize the importance of serum albumin levels, rather than urinary albumin excretion, as an independent predictor of nocturnal systolic dipping, at least in non-diabetic essential hypertensive patients with moderate proteinuria.

## INTRODUCTION

Deterioration of the circadian blood pressure (BP) rhythm, particularly a non-dipper circadian BP pattern, represents a risk factor for target organ damage and worsening of cardiovascular and renal parameters in patients with essential hypertension 1,2.

Ambulatory BP monitoring (ABPM), which provides data regarding changes in BP during daily activities 3, has been considered superior to office measurements of BP because ABPM identifies nocturnal declines in BP levels and has higher consistency with target organ damage, thus predicting cardiovascular morbidity and mortality^4^-^8^.

The limited amount of available data regarding the association between serum albumin levels and circadian BP rhythm indicates a correlation between less BP dipping and low serum albumin levels among the elderly 9, hemodialysis patients 10, patients with chronic kidney disease (CKD) 11,12 and hypertensive-naïve or nephrotic, non-diabetic patients^13^. Notably, the sodium retention and high sodium sensitivity caused by impaired daytime natriuresis have been shown to be associated with a loss of nocturnal decline in BP among patients with essential hypertension to provide compensatory pressure natriuresis during the nighttime 14-17. Moreover, the mechanism underlying the relationship between serum albumin and nighttime BP decline, the crucial predictor of cardiovascular events through vascular atherosclerosis, has not yet been elucidated 9.

Similarly, although numerous recent studies have focused on the relationship between urinary albumin excretion (UAE) and the loss of nocturnal BP decline, which are two notable risk markers of cardiovascular mortality and morbidity as well as end-stage CKD in hypertensive patients 13,18,19, it remains unclear whether albuminuria is a cause or a consequence of impaired circadian BP rhythm 13.

Moreover, given the inverse relationship between serum and urinary albumin levels, the presence of albuminuria concomitant with a loss of nighttime BP decline has been found to inevitably cause hypoalbuminemia in patients with non-diabetic nephropathy or glomerulonephritis 13. Therefore, the present study was designed to evaluate the relationship between serum albumin levels and 24-h ABPM recordings in non-diabetic essential hypertensive patients.

## MATERIALS AND METHODS

### Study population

This retrospective single-center study included 354 patients (mean [SD] age: 55.5 [14.3] years, 50% females) with a diagnosis of essential hypertension who received follow-up care, including 24-h ABPM recordings, at our clinic between 2013 and 2015. The patients were divided into 4 groups based on serum albumin levels: quartile 1 (Q1; serum albumin <4.1 g/dL), quartile 2 (Q2; serum albumin ≥4.1<4.4 g/dL), quartile 3 (Q3; serum albumin ≥4.4<4.6 g/dL) and quartile 4 (Q4; serum albumin ≥4.6 g/dL).

Patients with thyroid function disorders, secondary hypertension, diabetes mellitus, chronic liver disease, acute infection or sleep disorders were excluded from the study.

The study was conducted in full accordance with local Good Clinical Practice (GCP) guidelines and current legislation, and permission for the use of patient data for publication purposes was obtained from our institutional ethics committee.

### Study parameters

Data regarding patient demographics, body mass index (BMI; kg/m^2^), antihypertensive medication history, urinalysis (proteinuria [gr/24 h]) and blood biochemistry findings (urea [mg/dL]; creatinine [mg/dL]; e-GFR [mL/min], uric acid [mg/dL], sodium [mmol/L], potassium [mmol/L], calcium [mg/dL], phosphorus [mg/dL], CaxP product, intact parathormone [PTH; pg/mL], hemoglobin [g/dL], total cholesterol [mg/dL], triglycerides [mg/dL], low-density lipoprotein cholesterol [LDL-c; mg/dL], high-density lipoprotein cholesterol [HDL-c; mg/dL], albumin [g/dL] and ferritin [ng/mL]) were obtained from the medical records of each patient. The 24-h, nighttime and daytime ABPM values of systolic and diastolic BP; systolic and diastolic dipping status (% of dippers and non-dippers); and average amount of nocturnal dipping were recorded in all patients. Blood biochemistry findings, BMI and antihypertensive treatment were evaluated with respect to dipping status (dippers *vs*. non-dippers), whereas 24-h ABPM recording and average nocturnal dipping were evaluated with respect to quartiles of serum albumin levels (Q1-4) and dipping status. The correlation between serum albumin levels and nocturnal systolic and diastolic dipping was evaluated, and correlates of average nocturnal systolic dipping were determined via a linear regression model with age; serum levels of albumin, creatinine and hemoglobin; and 24-h urinary protein levels as the variables.

Serum albumin levels were measured with an ECLIA test performed using a Cobas E601 analyzer (Roche Diagnostics, MA, USA).

### ABPM recordings

A portable noninvasive recording device (Model 90217A-1, Spacelabs, Hertford, United Kingdom) was used to record 24-h ABPM values. All patients were graded according to overall, nighttime and daytime systolic and diastolic BP levels. The 24-h ABPM values were obtained via BP readings set at 20-minute intervals (06:00 AM – 24:00 PM) and at 30-minute intervals (24:00 PM – 06:00 AM). The patients were asked to continue with their normal daily routine and to record the times at which they went to sleep and awakened. The data were transferred to a computer and loaded into ABPM report management system software for the final analysis using the ABPM-FIT program (University of Heidelberg, Germany, version 2.2). For each 24-h measurement, the mean systolic and diastolic BP levels were evaluated separately for the daytime and nighttime periods. Daytime and nighttime arterial hypertension were diagnosed according to the ESH/ESC guidelines 20 (daytime: systolic BP ≥140 mmHg and/or diastolic BP ≥90 mmHg; nighttime: systolic BP >120 mmHg and diastolic BP >70 mmHg). The non-dominant arm was the site of cuff placement for ABPM. The ambulatory monitor had to agree with a mercury column sphygmomanometer to within 7 mmHg, and patients with failure of ≥25% of BP recordings on a daily basis were not included in the final analysis.

### Definitions of dippers and non-dippers

Time in bed was measured based on a diary maintained by the patients that documented the exact times of getting into and arising from bed. The average BP for the time in bed (termed nighttime BP) was calculated from the ambulatory monitoring data. The daytime BP was defined as the BP during the remainder of the 24-h period. The percentage decline in the nighttime BP was calculated as follows: 100 X (1 - mean nighttime systolic BP / mean daytime systolic BP). Patients with a decline in nighttime BP of less than 10% were considered non-dippers 7.

### Statistical analysis

The statistical analysis was performed using IBM SPSS Statistics (IBM Corp. Released 2012, IBM SPSS Statistics for Windows, Version 21.0. Armonk, NY: IBM Corp). A Chi-square (χ^2^) test was used for comparisons of categorical data. Student’s t-test was used for the analysis of parametric variables. Comparisons of albumin quartile groups were performed via one-way ANOVA. Spearman’s correlation analysis was performed to determine the relationship between serum albumin levels and dipping status, and correlates of average nocturnal systolic dipping were determined via a linear regression model with age; serum levels of albumin, creatinine and hemoglobin; and 24-h urinary protein levels as the variables. The data were expressed as the means (SD), percentages (%) and 95% confidence intervals (CIs), as appropriate. *P*<0.05 was considered statistically significant.

## RESULTS

### Baseline characteristics and 24-h ABPM recordings in dippers *vs*. non-dippers

Among the 354 patients, 95 (26.8%) were determined to be dippers, and 259 (73.2%) were non-dippers. No significant differences were noted between the dippers and non-dippers in terms of patient characteristics, BMI, antihypertensive medication or overall 24-h or daytime systolic and diastolic ABPM recordings. Compared with dippers, the non-dippers had significantly higher mean (SD) nighttime systolic (152.3 [23.9] mmHg *vs*. 130.8 [17.6] mmHg, *p*<0.001) and diastolic BP (82.3 [15.9] mmHg *vs*. 71.8 [11.7] mmHg, *p*<0.001), whereas they had significantly lower average nocturnal systolic (0.3 [6.6] mmHg *vs*. 15.2 [4.8] mmHg, *p*<0.001) and diastolic dipping (4.2 [8.6] mmHg *vs*. 18.9 [7.0] mmHg, *p*<0.001) (Table 1).

Compared with dippers, the non-dippers had significantly lower mean (SD) serum albumin levels (4.2 [0.3] *vs*. 4.4 [0.4] g/dL, *p*<0.001); otherwise, the two groups had similar laboratory results, including proteinuria (Table 2).

### 24-h ABPM recordings according to quartiles of serum albumin levels

The serum albumin quartiles Q1, Q2, Q3 and Q4 included 71 (20.1%), 100 (28.2%), 78 (22.0%) and 105 (29.7%) patients, respectively. Compared to the patients in Q1, the ABPM recordings of the other groups revealed significantly lower values as follows: mean (SD) 24-h systolic BP in Q2 (157.8 [19.0] mmHg *vs*. 148.7 [19.6] mmHg, *p*<0.005) and Q4 (157.8 [19.0] mmHg *vs*. 149.6 [20.0] mmHg, *p*<0.05); 24-h diastolic BP in Q2 (89.1 [15.2] mmHg *vs*. 81.6 [11.5], *p*<0.01); daytime diastolic BP in Q2 (89.8 [15.1] mmHg *vs*. 83.3 [12.0] mmHg, *p*<0.05); nighttime systolic BP in Q2, Q3 and Q4 (158.6 [23.1] mmHg *vs*. 143.8 [22.5] mmHg, 145.7 [27.9] mmHg and 141.3 [21.6] mmHg, respectively; *p*<0.001 for each); and nighttime diastolic BP in Q2, Q3 and Q4 (87.0 [17.5] mmHg *vs*. 76.0 [12.1] mmHg, 78.6 [16.4] mmHg and 78.3 [15.0] mmHg, respectively; *p*<0.001 for each) (Table 3).

### Average nocturnal systolic and diastolic dipping according to quartiles of serum albumin levels

**Figure 1:**
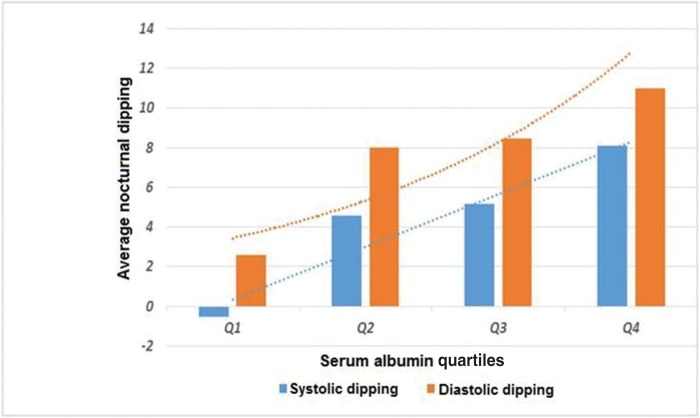
Average nocturnal dipping according to quartiles of serum albumin levels. Q1: serum albumin <4.1 g/dL; Q2: serum albumin ≥4.1<4.4 g/dL; Q3: serum albumin ≥4.4<4.6 g/dL; Q4: serum albumin ≥4.6 g/dL.

Compared with the patients in serum albumin quartile Q1, the patients in Q3 and Q4 had significantly higher mean (SD) nocturnal systolic dipping (-0.36 [8.42] mmHg *vs*. 4.79 [9.89] mmHg and 7.16 [8.78] mmHg, respectively; *p*<0.001 for each), and the patients in Q4 had significantly higher nocturnal diastolic dipping (3.48 [10.32] mmHg *vs*. 11.14 [10.25] mmHg, *p*<0.01) (Table 4,Figure 1).

### Correlation between serum albumin levels and nocturnal dipping

**Figure 2:**
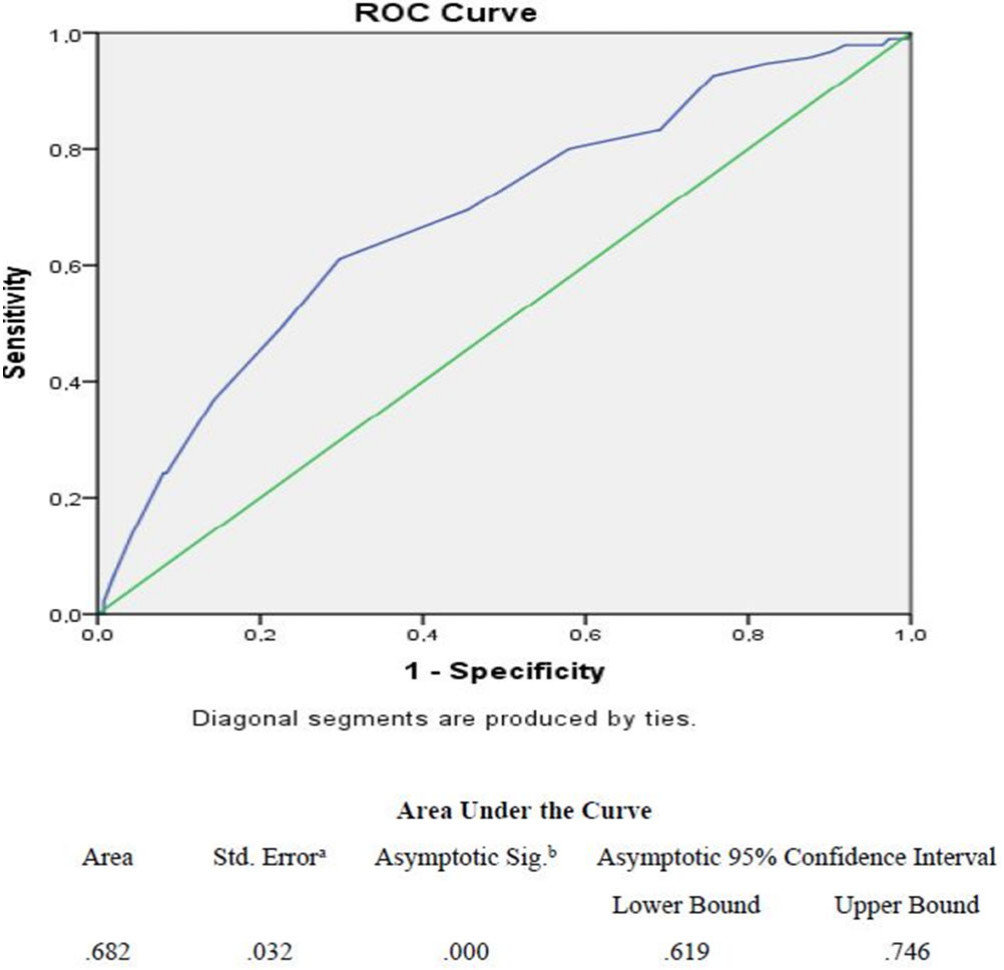
Receiver operating characteristic-curve analysis for sensitivity, specificity and cut-off values of the relationship between serum albumin levels and systolic dipping.

Significant positive correlations were noted between serum albumin levels and both systolic (r=0.297, *p*<0.001) and diastolic dipping (r=0.265, *p*<0.001) (Table 5 and Figure 2).

### Linear regression analysis of correlates of average nocturnal systolic dipping

The linear regression analysis revealed that for each one-unit increase in serum albumin, the average nocturnal dip in systolic BP increased by 0.17 mmHg (*p*=0.033), even after adjustments for age, serum creatinine, serum hemoglobin and 24-h urinary protein levels (Table 6).

## DISCUSSION

Our findings in a retrospective cohort of essential hypertensive patients revealed lower serum albumin levels in non-dippers than dippers. Moreover, lower average nocturnal dipping and thus higher nocturnal systolic and diastolic ABPM values were observed in patients with lower (<4.1 g/dL) serum albumin levels. Along with the positive correlation between albumin levels and average nocturnal systolic and diastolic dipping, a decrease in serum albumin was shown to be an independent predictor of lower average nocturnal systolic dipping.

The association between serum albumin levels and the loss of nocturnal BP decline has been reported in several studies of elderly patients^9^, hemodialysis patients 10, patients with CKD 11,12 and hypertensive-naïve or nephrotic non-diabetic patients 13.

A previous study of the effects of nutritional parameters on nocturnal BP in Turkish hemodialysis patients revealed an association between malnutrition and deterioration in the circadian BP rhythm 10. The malnutrition score was positively correlated with nighttime systolic BP, mean nighttime BP and mean 24-h arterial BP as well as with reductions in serum albumin and anthropometric indices 10. Therefore, low serum albumin levels and hypervolemia have been suggested to contribute to the association between impaired nutritional status and elevated nighttime BP 10.

In another study of 778 subjects including hypertensive naïve non-diabetic subjects (n=231), subjects without proteinuria (n=441) and non-diabetic nephrotic patients (n=37), nighttime BP decline was found to be associated with marked changes in both urinary and serum albumin levels 13.

In our study population, non-dipping status was associated with significantly lower serum albumin compared with dipping status and low albumin levels predicted lower average nocturnal systolic dipping. However, no significant correlation was noted between the amount of proteinuria and dipping status and moderate proteinuria was evident in both dipper and non-dipper hypertensive patients.

Serum albumin has been reported to be an independent predictor of loss of nocturnal BP decline even in subjects with no albuminuria or dipstick-positive proteinuria 13. In non-diabetic subjects, a significant loss of nighttime BP decline preceding the emergence of massive albuminuria and hypoalbuminemia was normalized following the correction of hyperalbuminuria and hypoalbuminemia^13^.

Given that hyperalbuminuria and hypoalbuminemia are inseparable and usually coexisting conditions, it has been considered difficult to evaluate the relationship between serum albumin levels and circadian BP patterns without considering albuminuria 13. Our findings indicate that serum albumin levels, rather than albuminuria, are a significant determinant of nighttime BP decline, at least among non-diabetic essential hypertensive patients with moderate proteinuria. Similarly, an increase in serum albumin to >3 g/dL was reported to be associated with a significant decrease in sleeping/waking mean BP in non-diabetic nephrotic patients, whereas lowering urinary albumin/creatinine to <300 mg/g was shown to be ineffective 13.

Notably, a disrupted 24-h BP rhythm has been suggested to precede microalbuminuria, whereas it is considered unlikely that microalbuminuria predisposes a patient to pathophysiological alterations that lead to a loss of nocturnal BP decline per se 13. These findings imply a potential impact of a greater burden of long-term traditional cardiovascular risk factors, at least in non-diabetic hypertensive patients 13.

In fact, a non-dipper circadian BP pattern was shown to be associated with subsequent microalbuminuria 2,21, which is a well-recognized marker of adverse cardiovascular outcomes in hypertensive subjects 22,23. Moreover, a close relationship between a diurnal rhythm of BP and albumin excretion has been suggested in patients with essential hypertension^21^.

Sodium retention and high sodium sensitivity were reported to cause a loss of nocturnal BP decline among patients with essential hypertension 15,16. Impaired daytime natriuresis has been suggested to be associated with elevations in nocturnal BP to facilitate pressure natriuresis and thus to compensate for the diminished natriuresis during the day 14,16,17. In this regard, given the association between hypoalbuminemia and low plasma oncotic pressure and shifting of sodium and fluid from the intravascular circulation to the extravascular space 14, the lower serum albumin levels among non-dipper compared with dipper patients in our study might also be associated with compensatory natriuresis.

However, although 73.0% of the hypertensive patients in our cohort were non-dippers, moderate proteinuria was evident regardless of dipping status.

Nonetheless, it should be noted that a debate continues regarding the common pathogenic mechanisms between UAE and circadian BP, with inconsistent findings regarding the role of a non-dipping pattern in the appearance of unfavorable UAE and regarding the value of absolute BP levels or dipping/non-dipping status in predicting UAE 21.

As an independent predictor of diabetic nephropathy and impaired renal function 24 and an indicator of inflammatory process of atherogenesis via its negative correlation with C-reactive protein levels 25, low serum albumin has been considered to play a key role in systemic vascular damage 13. Hypoalbuminemia, even a decrease of only 0.2 g/dL, has been suggested to be closely associated with an increased risk of cardiovascular events 26,27.

The significantly lower nighttime systolic and diastolic BP in the Q2, Q3 and Q4 serum albumin quartiles in our cohort suggest that a decline in albumin levels beyond 4.1 g/dL appears to increase the likelihood of non-dipping status in patients with essential hypertension.

A decrease in serum albumin was shown to be an independent predictor of lower average nocturnal systolic dipping and thus a nocturnal systolic non-dipping pattern, in our cohort. This finding appears to agree with reports that nighttime BP is more closely associated with the albumin-to-creatinine ratio (ACR) compared with daytime and 24-h BP 13.

Loss of nighttime BP decline has been shown to be a crucial risk marker for vascular atherosclerosis and cardiovascular events and an independent risk factor for overt CV disease 13,28-30. Along with the superiority of systolic over diastolic BP non-dipping in the prediction of target organ damage in essential hypertension 31, hypertensive nocturnal dippers were reported to exhibit a worse profile than normotensives regarding pulse wave velocity, ACR and hs-CRP 32. The identification of serum albumin levels as an independent predictor of nocturnal systolic non-dipping in our cohort supports the idea that serum albumin levels alone may provide sufficient data on systolic non-dipping and, thus, indices of target organ damage and cardiovascular risk 13,31.

Certain limitations of this study should be considered. First, due to the retrospective single-center design, establishing temporality between cause and effect and generalizing our findings to the overall essential hypertensive population may be difficult. Second, although no significant association was noted between dipping status and the type of hypertensive agents used by patients, we cannot exclude the possibility that the maintenance of treatment during ABPM altered the relationship between BP and the study outcomes. The limited data on surrogate markers of target organ damage and cardiovascular risk in hypertensive subjects is another limitation; the availability of such markers would extend the knowledge achieved in the current study. Despite these limitations, given the paucity of solid information available in this area, our findings represent a valuable contribution to the literature.

In conclusion, our findings indicate an association between serum albumin levels and the deterioration of circadian BP rhythm among essential hypertensive patients, with a non-dipper pattern identified in more than two-thirds of the patients. Our findings emphasize the importance of serum albumin levels, rather than UAE, as an independent predictor of nocturnal systolic dipping, at least in non-diabetic essential hypertensive patients with moderate proteinuria. Larger prospective ABPM studies addressing the relationship between circadian BP rhythm and serum and urinary albumin among hypertensive patients with massive albuminuria or hypoalbuminemia are needed, as are advanced biochemical markers of target organ damage and cardiovascular risk.

## AUTHOR CONTRIBUTIONS

Ahbap E, Sakaci T, Kara E and Sahutoglu T designed the study, collected and analyzed the data and wrote the manuscript. Basturk T and Koc Y were involved in designing the study and collecting the data. Sevinc M and Kara E performed the statistical analysis and collected the data. Akgol C, Ucar ZT, Kayalar AO and Bayraktar F collected the data. Unsal A was responsible for final editing of the manuscript.

## Figures and Tables

**Table 1:** Baseline characteristics and 24-h ambulatory blood pressure monitoring recordings in dippers vs. non-dippers.

Patient characteristics	Dippers (n=95)	Non-dippers (n=259)	*p* value^a^
Age (years), mean (SD)	53.4 (15.5)	56.3 (13.8)	0.092
Gender (M/F)	47/48	130/129	1.000
BMI (kg/m^2^), mean (SD)	30.9 (4.8)	30.1 (5.7)	0.554
**Antihypertensive medication**	**%**	**%**	
ACE inhibitor	36.7	33.3	0.687
Angiotensin receptor blocker	32.6	28.8	0.547
Beta-blocker	18.4	16.8	0.773
Calcium channel blocker	12.6	16.4	0.476
Diuretics	21.3	19.8	0.871
**24-h ABPM recordings (mmHg)**	**Mean (SD)**	**Mean (SD)**	
**Overall**			
24-h systolic BP	148.2 (20.1)	152.6 (20.9)	0.078
24-h diastolic BP	84.6 (13.1)	85.0 (14.8)	0.822
**Daytime**			
Systolic BP	154.1 (20.7)	152.7 (20.5)	0.590
Diastolic BP	89.0 (13.9)	85.8 (15.0)	0.077
**Nighttime**			
Systolic BP	130.8 (17.6)	152.3 (23.9)	**<0.001**
Diastolic BP	71.8 (11.7)	82.3 (15.9)	**<0.001**
**Average nocturnal dipping**			
Systolic	15.2 (4.8)	0.3 (6.6)	**<0.001**
Diastolic	18.9 (7.0)	4.2 (8.6)	**<0.001**

ABPM: ambulatory blood pressure monitoring; ACE: angiotensin-converting enzyme; BP: blood pressure; BMI: body mass index.

a Chi-square (χ2) test

b Student's t-test

**Table 2:** Laboratory findings in dipper and non-dipper patients.

	Dippers (n=95)	Non-dippers (n=259)	
Laboratory parameter	Mean (SD)	Mean (SD)	*p* value^a^
Urea (mg/dL)	50.3 (33.2)	55.8 (36.1)	0.153
Creatinine (mg/dL)	1.49 (1.16)	1.75 (1.67)	0.167
e-GFR (mL/dk)	62.1 (34.1)	56.0 (35.3)	0.557
Uric acid (mg/dL)	5.9 (1.6)	6.2 (1.6)	0.268
Sodium (mmol/L)	139.5 (3.6)	139.8 (3.1)	0.429
Potassium (mmol/L)	4.5 (0.5)	4.7 (0.5)	0.070
Calcium (mg/dL)	9.4 (0.5)	9.3 (0.5)	0.054
Phosphorus (mg/dL)	3.7 (0.8)	3.6 (0.8)	0.917
CaxP product	34.7 (7.9)	34.2 (7.1)	0.590
Intact PTH (pg/mL)	97.0 (88.3)	110.2 (106.3)	0.413
Hemoglobin (g/dL)	13.3 (1.8)	13.0 (1.9)	0.153
Total cholesterol (mg/dL)	202.0 (39.8)	201.6 (44.8)	0.943
Triglycerides (mg/dL)	149.3 (76.0)	171.4 (131.5)	0.163
LDL-cholesterol (mg/dL)	118.7 (37.8)	120.9 (39.6)	0.674
HDL-cholesterol (mg/dL)	51.1 (19.5)	51.7 (52.1)	0.927
Albumin (g/dL)	4.4 (0.4)	4.2 (0.3)	**<0.001**
Ferritin (ng/mL)	135.8 (211.0)	113.1 (127.6)	0.279
Proteinuria (gr/24 h)	0.79 (1.15)	1.13 (1.82)	0.091

CaxP: calcium x phosphorus; e-GFR: estimated glomerular filtration rate; HDL: high-density lipoprotein; LDL: low-density lipoprotein; PTH: parathormone.

a Dipper *vs*. non-dipper; Student’s t-test

**Table 3:** 24-h Ambulatory blood pressure monitoring recordings according to quartiles of serum albumin levels.

		ABPM recordings					
		Overall-24 h		Daytime (mmHg)		Nighttime (mmHg)	
	Serum albumin levels	Systolic BP	Diastolic BP	Systolic BP	Diastolic BP	Systolic BP	Diastolic BP
**Albumin quartiles**	Mean (SD)	Mean (SD)	Mean (SD)	Mean (SD)	Mean (SD)	Mean (SD)	Mean (SD)
Q1 (n=71)	3.6(0.5)	157.8(19.0)	89.1(15.2)	157.6(18.7)	89.8(15.1)	158.6(23.1)	87.0(17.5)
Q2 (n=100)	4.2(0.1)***	148.7(19.6)*	81.6(11.5)**	150.5(19.3)	83.3(12.0)*	143.8(22.5)***	76.0(12.1)***
Q3 (n=78)	4.4(0.2)***	151.5(23.8)	83.9(15.7)	153.1(23.2)	85.7(16.1)	145.7(27.9)***	78.6(16.4)***
Q4 (n=105)	4.7(0.1)***	149.6(20.0)*	85.8(14.6)	152.5(20.5)	88.5(15.3)	141.3(21.6)***	78.3(15.0)***

BP: blood pressure; SD: standard deviation; Q: quartile

Q1: serum albumin <4.1 g/dL

Q2: serum albumin ≥4.1<4.4 g/dL

Q3: serum albumin ≥4.4<4.6 g/dL

Q4: serum albumin ≥4.6 g/dL

* *p*<0.05,

** *p*<0.01

*** *p*<0.001 compared to Q1; ANOVA and *post-hoc* Tukey tests

**Table 4:** Average nocturnal systolic and diastolic dipping according to quartiles of serum albumin levels.

Albumin quartiles	Nocturnal dipping, mean (SD)	
	Systolic	Diastolic
**Q1 (n=71)**	-0.36(8.42)	3.48(10.32)
**Q2 (n=100)**	4.44(7.80)	8.52(10.12)
**Q3(n=78)**	4.79(9.89)**	8.20(9.96)
**Q4 (n=105)**	7.16(8.78)**	11.14(10.25)*

Q: quartile; Q1: serum albumin <4.1 g/dL; Q2: serum albumin ≥4.1<4.4 g/dL; Q3: serum albumin ≥4.4<4.6 g/dL; Q4: serum albumin ≥4.6 g/dL.

* *p*<0.01

** *p*<0.001 compared to Q1; ANOVA and *post-hoc* Tukey tests

**Table 5:** Correlations between serum albumin levels and nocturnal dipping.

		Albumin	Systolic dipping	Diastolic dipping
Albumin	N	354	354	354
	r	1.000	0.297	0.265
	*p*	-	**<0.001**	**<0.001**
Systolic dipping	N	354	354	354
	r	0.297	1.000	0.831
	*p*	**<0.001**	-	**<0.001**
Diastolic dipping	N	354	354	354
	r	0.265	0.831	1.000
	*p*	**<0.001**	**<0.001**	-

Spearman’s correlation analysis; r=correlation coefficient.

Bold values indicate correlations significant at the 0.01 level (2-tailed).

**Table 6:** Linear regression analysis of correlates of average nocturnal systolic dipping.

Model (enter method)	Beta	*p* value	95% Confidence interval	
			Lower bound	Upper bound
Constant	----	0.423	-1.047	0.440
Age	-0.024	0.719	-0.005	0.003
Serum creatinine	-0.005	0.941	-0.055	0.051
Serum albumin	0.170	0.033	0.012	0.287
Serum hemoglobin	-0.014	0.852	-0.035	0.029
24-h urinary protein	-0.004	0.964	-0.041	0.039
